# Diabetic Mouse Model of Orthopaedic Implant-Related Staphylococcus Aureus Infection

**DOI:** 10.1371/journal.pone.0067628

**Published:** 2013-06-20

**Authors:** Arianna B. Lovati, Lorenzo Drago, Lorenzo Monti, Elena De Vecchi, Sara Previdi, Giuseppe Banfi, Carlo L. Romanò

**Affiliations:** 1 Cell and Tissue Engineering Laboratory, Gruppo Ospedaliero San Donato Foundation, Milan, Italy; 2 Laboratory of Clinical Chemistry and Microbiology, IRCCS Galeazzi Orthopaedic Institute, Milan, Italy; 3 Department of Biomedical Science for Health, University of Milan, Milan, Italy; 4 Dipartimento di Chirurgia Ricostruttiva e delle Infezioni Osteo-articolari, IRCCS Galeazzi Orthopaedic Institute, Milan, Italy; 5 Laboratory of Cancer Cachexia AIRC Start-Up, Oncology Department, Istituto di Ricerche Farmacologiche Mario Negri, Milan, Italy; 6 Laboratory of Experimental Biochemistry and Molecular Biology, IRCCS Galeazzi Orthopaedic Institute, Milan, Italy; Institut Pasteur, France

## Abstract

**Background:**

Periprosthetic bacterial infections represent one of the most challenging orthopaedic complications that often require implant removal and surgical debridement and carry high social and economical costs. Diabetes is one of the most relevant risk factors of implant-related infection and its clinical occurrence is growing worldwide. The aim of the present study was to test a model of implant-related infection in the diabetic mouse, with a view to allow further investigation on the relative efficacy of prevention and treatment options in diabetic and non-diabetic individuals.

**Methodology:**

A cohort of diabetic NOD/ShiLtJ mice was compared with non-diabetic CD1 mice as an in vivo model of S. aureus orthopaedic infection of bone and soft tissues after femur intramedullary pin implantation. We tested control and infected groups with 1×10^3^ colony-forming units of S. aureus ATCC 25923 strain injected in the implant site. At 4 weeks post-inoculation, host response to infection, microbial biofilm formation, and bone damage were assessed by traditional diagnostic parameters (bacterial culture, C-reactive protein and white blood cell count), histological analysis and imaging techniques (micro computed tomography and scanning electron microscopy).

**Results:**

Unlike the controls and the CD1 mice, all the diabetic mice challenged with a single inoculum of S. aureus displayed severe osteomyelitic changes around the implant.

**Conclusions:**

Our findings demonstrate for the first time that the diabetic mouse can be successfully used in a model of orthopaedic implant-related infection. Furthermore, the same bacteria inoculum induced periprosthetic infection in all the diabetic mice but not in the controls. This animal model of implant-related infection in diabetes may be a useful tool to test in vivo treatments in diabetic and non-diabetic individuals.

## Introduction

Post-operative bacterial infection is among the most challenging orthopaedic complications associated with primary (1–4%) and revision (>20%) prosthetic [1]–[3] and implant surgery [4]. Septic complications are the first and the third reason for knee and hip joint prosthesis failure, respectively, in the Unites States [5]. These severe complications frequently lead to implant removal and/or extensive surgical debridement and carry a high social and economical burden [6]–[8].

Diabetes mellitus is one of the most relevant risk factors for post-operative orthopaedic infections [9], [10]. Studies investigating the differences in infection response in diabetic and non-diabetic patients cite innate immune system defects and increased adherence of microorganisms to cells as the prime reason causes for the impaired ability of diabetics to fight infection [11], [12]. In diabetic patients receiving a prosthetic implant, the rate of infection is more than 10% higher than in non-diabetic subjects [13] and type 1 diabetes has been reported to be associated with a higher risk of post-operative complications after hip or knee arthroplasty as compared to type 2 diabetes [14]. In addition, diabetes induced neuropathy and vasculopathy play an additional role in the development of infections and relative high mortality [9].

As the world population ages, the incidence of diabetes mellitus and orthopaedic prosthetic surgery will increase in the coming years [15].

The most common pathogens of implant-related infections are opportunistic microorganisms, including methicillin-susceptible or resistant *Staphylococcus epidermidis* and *aureus* (80%), *Streptococcus spp.* and Gram-negative bacteria (20%). Typically, these bacteria are able to adhere to one another, forming a microbial assembling embedded in an extracellular matrix, the “biofilm”, that leads to persistent local (osteomyelitis) and/or systemic infections and retains a multifactorial tolerance to host immune cells and antibiotic treatments [16]–[18].

Although infections in diabetic patients are frequently polymicrobial, *S. aureus* and *epidermidis* account for the pathogens most often the cause of post-surgical infections, with many strains resistant to antibiotics, making the treatment of infections very problematic [17]. Patients with type 1 diabetes show more frequent colonization of the skin by *S. aureus* than non-diabetic and non-insulin dependent diabetic individuals [19]. Various diabetic animal models have been developed to study the pathogenesis of diabetes and its complications and to test novel diabetic treatments before clinical use [8]. The most common model for type 1 diabetes is the non-obese diabetic mouse (NOD/ShiLtJ) which spontaneously develops diabetes closely resembling the pathophysiology of the condition in humans [20]. In orthopaedics, however, diabetic animal models have been used only to investigate delayed bone fracture healing [21] or diabetic foot infections [10].

In this study, we compared the diabetic NOD/ShiLtJ mouse with the non-diabetic CD1 mouse as an *in vivo* model of *S. aureus* orthopaedic infection of bone and soft tissues after femur intramedullary pin implantation. To do this, we used conventional diagnostic parameters (bacterial culture, C-reactive protein and white blood count), histological findings and advanced imaging technologies in orthopaedic (micro-computed tomography [micro-CT] and scanning electron microscopy [SEM]) [22], [23] to assess host response to infection, microbial biofilm formation and bone damage in the healthy and the diabetic mice.

## Materials and Methods

### Ethics Statement

The whole study was approved by the Mario Negri Institute for Pharmacological Research (IRFMN) Animal Care and Use Committee (IACUC) (Permit N. 43_2013-B) which includes "ad hoc" members for ethical issues. Animals and their care were handled in compliance with institutional guidelines as defined in national (Law 116/92, Authorization n.19/2008-A issued March 6, 2008, by the Italian Ministry of Health) and international laws and policies (EEC Council Directive 86/609, OJ L 358. 1, December 12, 1987; Standards for the Care and Use of Laboratory Animals - UCLA, U. S. National Research Council, Statement of Compliance A5023-01, November 6, 1998). The animals were housed at the Institute's Animal Care Facilities that meet international standards; they were regularly checked by a certified veterinarian responsible for health monitoring, animal welfare supervision, experimental protocols and procedure revision.

### Experimental design

The diabetic mouse (NOD/ShiLtJ) model of staphylococcal orthopaedic implant-related infection was compared to the non-diabetic mouse (CD1) model of implant-related infection and to controls.

The mice were randomly assigned to the one of four experimental groups (n = 8 animals in each group):

Group I CD1 mice + 3 µl Phosphate Buffered Saline (control/PBS)

Group II CD1 mice + *S. aureus* 10^3^ Colony Forming Unit (CFU)/3 µl (infected/SA)

Group III NOD/ShiLtJ mice + 3 µl PBS (control/PBS)

Group IV NOD/ShiLtJ mice + *S. aureus* 10^3^ CFU/3 µl (infected/SA)

Table 1 reports the allocation of animals per group and the relative analysis.

**Table 1:** Allocation of animals per group and investigations.

INVESTIGATIONS	NUMBER OF ANIMALS							
	**1**	**2**	**3**	**4**	**5**	**6**	**7**	**8**
**Blood analysis**	x	x	x	x	x	x	x	x
**Micro-CT**	x	x	x	x	x			
**Histology**	x	x	x					
**Microbiology**					x	x	x	x
**SEM**				x				

### Preparation of *S. aureus* for inoculation into the joint space

*S. aureus* strain ATCC 25923 was used in this study. Bacteria were cultured at 37°C overnight onto Mannitol Salt Agar (BioMerieux, France). To prepare the inoculum, a selected colony was inoculated into Brain Heart Infusion Broth (BioMerieux) and incubated for 16 h at 37°C. The bacterial suspension was then washed twice and the obtained pellet was suspended in PBS to obtain a 0.5 McFarland turbidity (equal to about 1×10^8 ^CFU/mL), and further diluted to obtain a bacterial concentration of about 3.3×10^5 ^CFU/mL. The bacterial suspension was then serially diluted with sterile saline solution and counts were performed to check the bacterial inocula for the experiments. The bacterial suspension was used within 2 h and stored on ice until use.

### *In vivo* surgical procedures

Sixteen female CD1 14 week old mice (mean body weight 37.5 g) (Charles River, Italy) and sixteen female NOD/ShiLtJ type I diabetic 14 week old mice (mean body weight 23.5 g) (Jackson Laboratory) were used for the experiments. Blood glucose levels in the NOD/ShiLtJ mice were tested directly by Charles River to guarantee the diabetic status of the animals on delivery. The mice were maintained under specific pathogen-free conditions and fed with autoclaved food and water provided *ad libitum*. All pre-surgical and surgical procedures on the animals were performed under a laminar flow hood.

**Figure 1:**
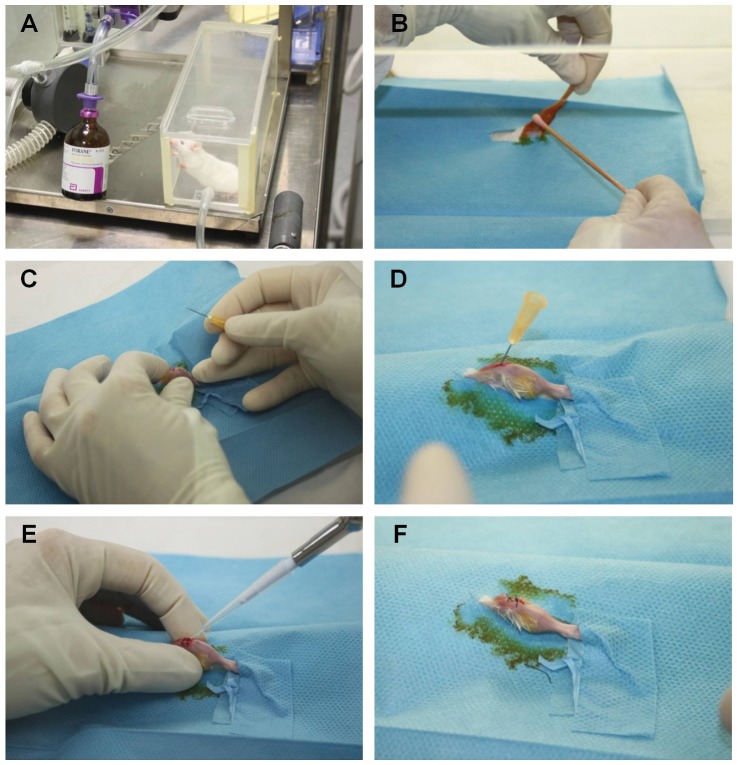
Mouse surgical procedures. Anesthesia chamber with isoflurane inhalation (A); skin preparation (B); skin incision over the right knee (C); introduction of a 25-gauge needle retrograde into the femoral medullary canal (D); inoculation of 3 µl of *S. aureus* (E) and closure of the surgical site with interrupted sutures (F).

The mice were anesthetized via inhalation of isoflurane (2%) (Fig. 1 A) and maintained with an intraperitoneal injection of ketamine chloride 80 mg/kg (Imalgene, Merial, Italy) and medetomidine hydrochloride 1 mg/kg (Domitor, Pfizer, Italy). All efforts were made to minimize suffering. After skin preparation by shaving and disinfection with alcohol and povidone iodine (Fig. 1 B), an incision was performed over the right knee (Fig. 1 C). The distal right femur was accessed through a medial parapatellar arthrotomy. After identifying the intercondylar notch, a 25-gauge needle (Ø 0.5 mm), used as implant, was inserted retrograde into the medullary canal of the femur to a depth of 7–10 mm and then cut so that 1 mm protruded into the joint space (Fig. 1 D). This surgical technique has been described elsewhere [24]. The metallic implant was left in the medullar canal of the femur for 28 days. In this study, we chose an intramedullary implant approach since it reproduces the serious infections seen in patients after total joint replacement. In the infected groups (II and IV), a volume of 3 µL of the bacterial suspension, corresponding to an inoculum of about 1×10^3 ^CFU/mouse, according to the literature [24], [25], was injected into the joint space on the protruding edge of the implant (Fig. 1 E) and the solution was allowed to spread throughout the medullary canal. In the sham-inoculated control groups (I and III), sterile PBS was injected as described above. The quadriceps-patellar complex was repositioned to the midline, and the incision was sutured with interrupted stitches (Ethilon 4-0, Ethicon, US) (Fig. 1 F). Micro-CT of the contralateral limb was used as the untreated healthy control for all animals.

Immediately after surgery, all animals received a one-shot injection of carprofen 5 mg/kg SC (Rimadyl, Pfizer, Italy) and ceftriaxone 60 mg/kg IM (Rocephin, Roche, Italy). Atipamezole 1 mg/kg (Antisedan, Pfizer, Italy) was administered subcutaneously to recover the animals from general anesthesia. The animals were then housed in separate cages under a heating lamp and monitored until the effects of anesthesia had worn off. About 24 h later, the animals were grouped four per cage, monitored daily for general status and welfare, and checked weekly to record data on body weight, clinical signs of infection, lameness, weight bearing, swelling, wound healing, pain and suffering. Pain was controlled with buprenorphine (0.1 mg/kg SC).

After 4 weeks, the mice were euthanized by CO_2_ inhalation to perform the investigations.

Surgical dissection was performed under a laminar flow hood and in sterile conditions; the soft tissues were inspected for gross appearance (signs of inflammation and infection), then stripped off, and the femurs were disarticulated at the hip joint to be removed.

### Blood collection and analysis

Blood samples for total white blood cells (WBC) count and C-Reactive Protein (CRP) (n = 32) determination were collected from the facial vein on day 0 and directly from the left ventricle immediately after sacrifice (day 28).

Facial vein blood draws were done with an 18G needle puncturing the skin slightly dorsally (1–2 mm) to the mandible angle to a shallow depth of approximately 1–2 mm and a volume of 200 µl of blood was collected by allowing the blood to drop directly into centrifuge tubes.

Heart blood was collected through a 21G needle inserted into the abdomen near the sternum and directed towards the heart; 500 µl of blood were obtained from the left ventricle.

The blood was immediately transferred to centrifuge tubes containing the appropriate anticoagulants for cell counting or into empty tubes for measuring serum CRP. EDTA anticoagulated blood samples for total WBC were processed on an automatic cell counter for human use (Sysmex XT-1800, Dasit). To measure CRP, the sera were serially diluted and the protein was measured with a latex immunoassay (CRP Vario, Abbott Diagnostics) according to the manufacturer’s instructions.

### Micro-CT imaging and data analysis

To evaluate periosteal, cortical and endosseous reaction, and the development of deformities, osteomyelitis and osteolysis, micro-CT analysis (n = 5 per group) were performed.

Immediately after sacrifice, the femurs were removed, placed into a culture dish and scanned with an Explore Locus micro-CT scanner (GE Healthcare, London, Ontario, Canada), without using contrast agents.

A micro-CT lower-resolution (Bin-2) protocol was performed using 80 kV voltage, 450 µA current with 300 msec exposure time per projection and 720 projections over 360° for a total scan time of approximately 24 minutes. The isotropic resolution of this protocol is 45 µm. The radiation dose was estimated to be 0.60 Gy. The reconstructed 3D images were viewed and analyzed using the Micro View image viewer (version 2.1.2; GE Healthcare) for the analysis of osteomyelitis. The images from each sample were binarized at identical thresholds to allow for unbiased identification of bone damage and osteolysis.

An image analysis approach was designed specifically to measure the outer bone volume of the femur and evaluate anatomical changes. Briefly, a volume of interest (VOI) around the hind limb was designed and the bone structure was separated from air and soft tissues with a global thresholding procedure. After defining the optimal threshold for bone, an isosurface volume rendering function (a ray cast method that renders the surfaces of objects of similar density and hides the remaining materials) was applied to visualize and determine the presence and extent of infection-induced osteolytic lesions and bone reactions.

Bone mineral density (BMD) was measured with micro-CT, which was calibrated during the first scan of each session using a phantom made of an epoxy-based resin that mimics hydroxyapatite and contains water and air inclusion. The phantom was placed in the field of view of the scanned specimen. Before volume reconstruction, the data were calibrated against the phantom using the reconstruction software. The BMD (mg/cc) was measured on the bone volume designed on the femoral bone by the Micro View bone analysis tool.

### Histological analysis

Femoral specimens (n = 3 per group) were dissected and fixed in 10% formalin overnight.

The bones were decalcified in Mielodec (Bio-Optica, Milan, Italy) for 4 days, dehydrated in 70% (vol/vol) ethanol, embedded in paraffin and cut into 5 µm sagittal sections. The slides were dried, deparaffinized and stained with haematoxylin and eosin (H&E) as per standard protocol to assess morphology and with Gram staining for bacterial examination.

Photomicrographs were captured using an Olympus IX71 light microscope and an Olympus XC10 camera.

The severity of inflammation and infection was evaluated on the basis of periosteal reaction, cortical bone and medullary canal alterations, according to the modified grading score proposed by Petty et al. [26] (Table 2).

**Table 2:** Histological classification by modified Petty et al. (1985).

	Periosteum	Cortex	Medullary canal
**0**	Absence of reaction	Small Haversian canals, subperiosteal resorption, absence of polymorpho-nucleated leukocytes	Absence of inflammatory cells or foci of intact polymorpho-nucleated leukocytes
**1**	Laminated with 1–2 layers	Occasional polymorph nucleated leukocytes in Haversian canals	Diffuse polymorpho-nucleated leukocytes
**2**	Sunburst type	Focally enlarged Haversian canals filled with granulation tissue, fragmented polymorpho-nucleated leukocytes and osteoclasts	Diffuse intact and fragmented polymorpho-nucleated leukocytes, several micro abscesses
**3**	Florid, sunburst type	Subperiosteal, endosteal and intracortical resorption, fragmented polymorpho-nucleated leukocytes and micro abscesses, many osteoclasts	Several micro- and macroabscesses in diffuse polymorpho-nucleated leukocytes

### Microbiological analysis

To evaluate bacterial bone colonization, bacteria were recovered from the bone samples (n = 4 per group) taken on day 28 from all animals under aseptic conditions. The specimens were placed in sterile tubes and weighed, then cut into small pieces, transferred to a sterile tube containing 1 mL of normal sterile saline and sonicated in an ultrasound bath (VWR, Italy) for 5 min at a frequency of 30 kHz and a power output of 300 W, at room temperature. After vortexing for 5 min, aliquots of the sonicated fluid were seeded on Mueller-Hinton agar (BioMerieux) and incubated for 16 h at 37°C. After incubation, colonies resembling *S. aureus* were counted and the (Log CFU)/g bone was determined. In particular, colonies were assessed for Gram stain and catalase tests. Gram-positive catalase positive cocci were subcultured onto Mannitol Salt Agar and checked for coagulase production. Mannitol positive coagulase positive colonies were identified as *S. aureus* and counted. The detection limit was 1.3 (Log CFU)/g of bone. A single microbiologist blinded to the specimen groups conducted all microbiological analyses.

### SEM analysis

After explantation, one sample per group was fixed in 2.5% paraformaldehyde and 2.5% glutaraldehyde in 0.1 M Na-Cacodylate buffer (pH 7.4). After fixation, the samples were rinsed with Na-Cacodylate buffer and fixed for 1h in OsO_4_ (1% in Na-Cacodylate buffer), then samples dehydrated in ethanol, mounted on aluminum stubs and sputter-coated with gold using a SEMPREP 2 Sputter Coater *(*Nanotech Ltd). Observations were performed with a LEO 1400 EVO Scanning Electron Microscope (Zeiss) mixing secondary (SE) and backscattered electrons (BSE) detectors. Images were acquired at 10 kV at a working distance of 7 mm.

### Statistical analysis

Comparisons between groups were analyzed with one-way analysis of variance (ANOVA) (Instat 2.0; Graphpad Software, San Diego, CA). Comparisons between groups and time points were analyzed with two-way ANOVA. When significant differences were detected, post hoc comparisons of means were performed using Bonferroni’s procedure. All data are expressed as means ± standard error (SEM). Values of *P*<0.05 were considered statistically significant.

## Results

### Gross appearance and clinical data

The NOD/ShiLtJ mice developed early diabetes with moderate glycosuria and non-fasting plasma glucose levels > 130 mg/dl at 14 weeks of age.

**Figure 2:**
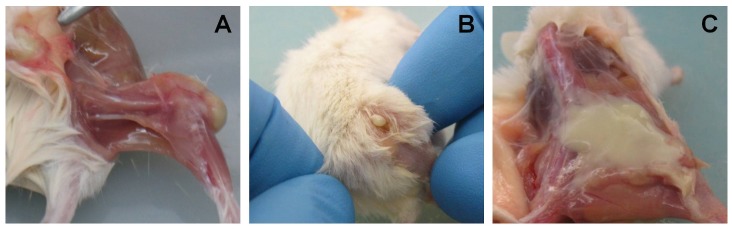
Gross appearance at explantation. Draining abscess in the knee joint (A) and the hip joint (B), involving the subcutaneous tissue in the mice in group IV (C).

The CD1 mice inoculated with 10^3^ CFU *S. aureus* (group II) showed mild lameness 7 days after implantation which promptly resolved the week after, whereas the NOD/ShiLtJ mice inoculated with 10^3^ CFU *S. aureus* (group IV) developed persistent lameness, restricted movement or avoided weight bearing and vacillated in use of the treated limb. Also noted were severe signs of infection, with erythema and swelling of soft tissues, knee and hip joint abscesses, fistulae, sometimes purulent exudates on compression, and loss of proprioception starting the first week post-inoculation. Two animals with severe signs of infection received additional treatment with buprenorphine for pain control. These observations were confirmed on dissection and gross examination after sacrifice 4 weeks after implantation. Group IV showed draining abscesses (Fig. 2 A, B) or subcutaneous abscesses at the knee and hip joints (Fig. 2 C). No control groups (I and III) showed any sign of infection. None of the animals was lost due to infection during the 4 weeks of observation.

**Figure 3:**
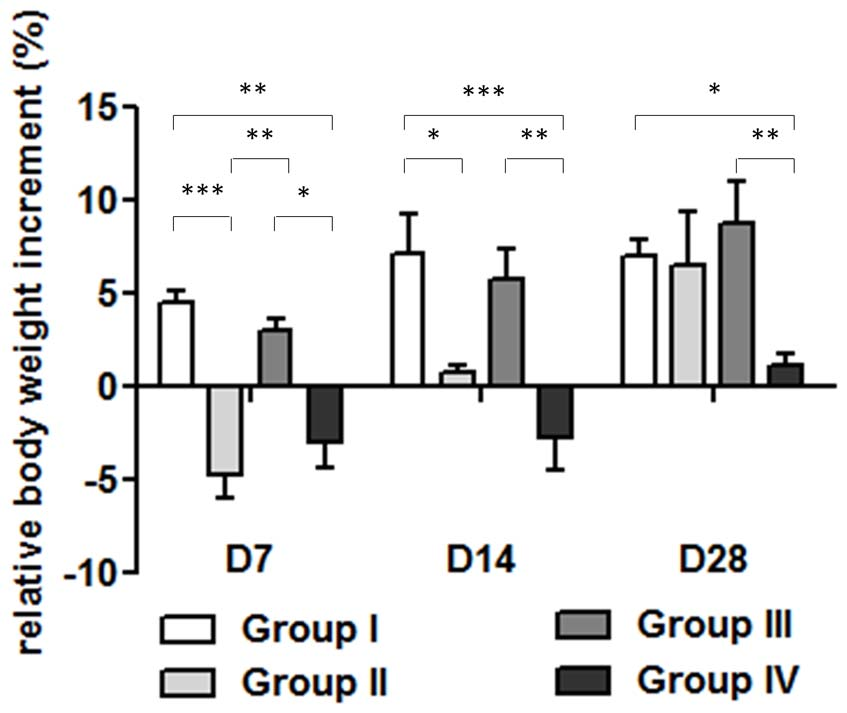
Relative changes in body weight. The histogram shows the relative changes in body weight in the experimental groups over time. A significant weight loss was noted in both groups II and IV versus their controls at 7 days after surgery. Group II recovers body weight starting from the second week after surgery. Differently, group IV regained body weight slowly. Comparisons between groups and time points were analyzed with two-way ANOVA, and Bonferroni’s post-hoc. Statistical significance for P<0.001 (***), <0.01 (**), <0.05 (*); n = 8.

Infection in the NOD/ShiLtJ mice (group IV) was associated with a marked and persistent loss in body weight by day 7 post-infection as compared to their respective controls (group III). Group II showed a similar loss in body weight at 7 days after implantation, but they recovered quicker than the NOD/ShiLtJ mice and a continued to regain weight (Fig. 3). The histogram in Fig. 3 reports the percentage changes in body weight versus baseline (day of inoculation/implantation).

### Blood analysis

**Figure 4:**
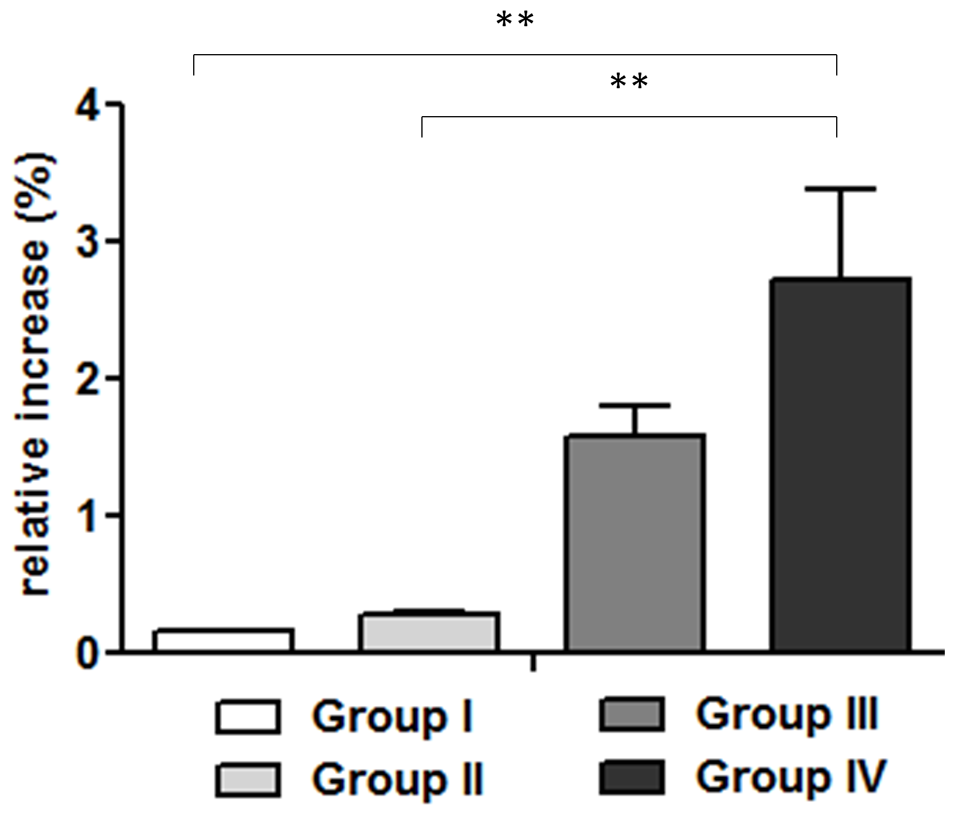
Relative increase in total WBC. On day 28, relative increases in WBC were significantly higher in group IV than in the other groups (P<0.01**; n = 8). Comparisons between groups were analyzed with one-way ANOVA, and Bonferroni’s post-hoc.

Basal total WBC was higher in the NOD/ShiLtJ mice than in the CD1 mice in either control group (I and III). After 28 days from surgery, the increase in total WBC count was higher in group IV than in group III. In particular, group IV had higher WBC counts as compared to their respective controls (group III) and groups I and II (Fig. 4).

The serum CRP levels, both at the day of surgery (D0) and after 28 days, remained very low (1.24±0.06 µg/mL).

### Micro-CT imaging analysis

**Figure 5:**
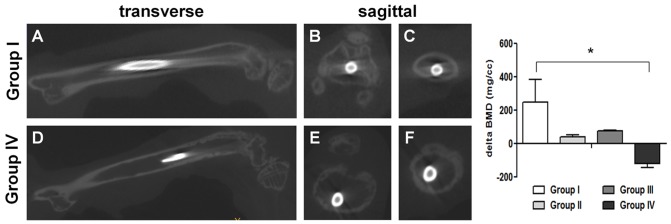
Representative micro-CT images and bone mineral density (BMD). Representative maximal intensity projection micro-CT images of the femurs with the pin implants in transverse (A, D) and sagittal (B, C, E, F) views in group I (upper panel) and group IV (lower panel). The upper panel shows the intact cortical bone profile without signs of infection (A), of the knee joint (B) and of the middle diaphysis section (C) in group I. The lower panel shows extensive bone loss, disruption of cortical integrity, cortical thinning and canal widening (D), acute septic arthritis of the knee joint (E) and bone loss in the middle diaphysis section (F) suggesting signs of established infection in group IV. The histogram shows a statistically significant difference in the BMD of group IV, as analyzed with one-way ANOVA and Bonferroni’s post-hoc (*P<0.05; n = 5).

Micro-CT examination confirmed that the pin was in place in all the animals (Fig. 5).

On micro-CT analysis, the controls (groups I and III) and the infected CD1 mice (group II) showed bone integrity and topography in cortical or endosteal bone (Fig. 5 A, B, C). In group IV, *S. aureus* produced extensive bone loss along the femoral metaphysis, disruption of cortical integrity, and bone remodeling with cortical thinning and canal widening (Fig. 5 D, E, F). In addition, signs of septic arthritis were present with disruption of cartilage and subchondral bone within the knee joint (Fig. 5 E).

The change in BMD for each group was analyzed by normalizing each value to that of the intact femur of a healthy CD1 mouse (1818.96 mg/cc). A decrease in BMD was noted in the infected NOD/ShiLtJ mice (group IV) as compared to group I, as shown in the histogram of Fig. 5.

### Histological analysis of bones and joints

To determine the location of inflammatory infiltrate and bacterial inoculum within the infected bones and joints, histological sections of tissues harvested from the *S. aureus*-inoculated (group II and IV) and the sham-inoculated control mice (group I and III) at 28 days were examined.

**Figure 6:**
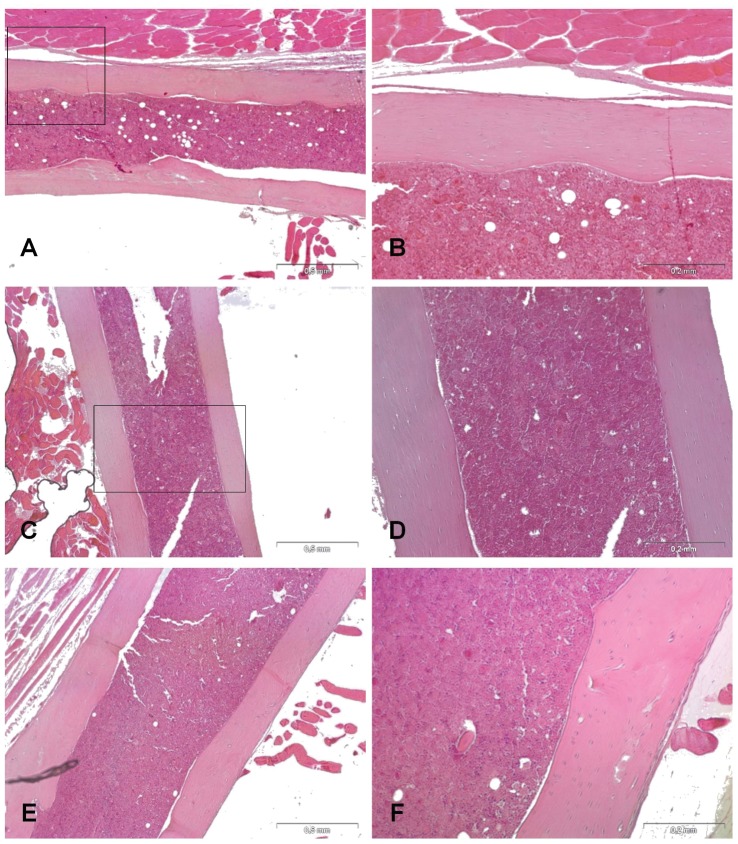
Histology of the femur in groups I and III and in group II. H&E staining of longitudinal sections of femurs. Note the absence of inflammatory cells in the medullary canal, bone resorption and periosteal reaction in group I (A, B), group III (C, D), and group II (E, F) 28 days after surgery. Left panel magnification 4X (scale bars 0.5 mm); right panel magnification 10X (scale bars 0.2 mm).

The samples obtained from groups I, III and II showed no or mild presence of inflammatory cells in the medullary canal which contained normally erythroid, and myeloid cells. No bone resorption or periosteal reactions were found in these samples (Fig. 6).

**Figure 7:**
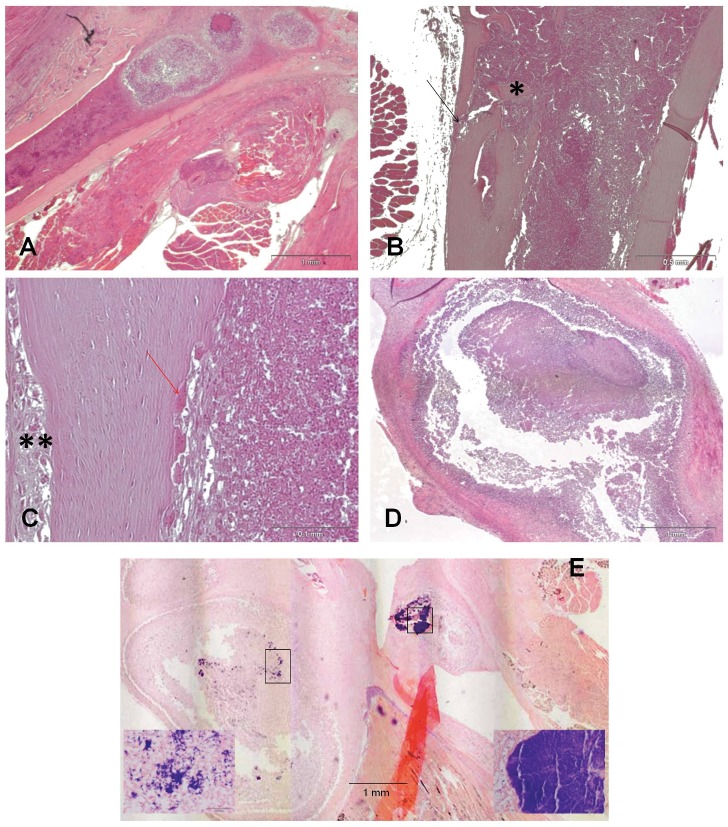
Histology of the femur and lymph node in group IV. H&E staining showing inflammatory cells and micro abscesses in the medullary canal (A, 2X, scale bar 1 mm); sequestrum formation (*) and fistula (black arrow) in the cortical bone (B, 4X, scale bar 0.5 mm); endosteal bone resorption, several osteoclasts (red arrow), and marked periosteal reaction (**) (C, 20X, scale bar 0.1 mm). A lymph node reaction is shown in image D (2X, scale bar 1 mm). Image E (2X, scale bar 1 mm) showing Gram-positive staining of bacterial clusters within a macroabscess of the knee joint capsule (left small box, 40X, scale bar 0.05 mm) and within a microabscess in the medullary canal (right small box, 40X, scale bar 0.05 mm).

In contrast, Gram-positive bacteria aggregates were detected in the medullary canal of the infected NOD/ShiLtJ mice (group IV) (Fig. 7 E), associated with a marked neutrophil infiltration and micro- and macro-abscesses in the medullary canal and within the joint capsule in the knee or hip joints (Fig. 7 A). New bone formation and endosseous cortical bone resorption by osteoclasts accompanied by enlargement of the medullary canal were interpreted as signs of osteomyelitis. In group IV, polymorpho-nucleated leukocytes and other inflammatory cells were present (Fig. 7 B), as were severe periosteal reaction and fibrosis (Fig. 7 C) and inguinal lymph node enlargement (Fig. 7 D). Table 3 reports the histological scores of the four groups.

**Table 3:** Histological scores (n = 3).

Group	Sample	Periosteum	Cortex	Medullary canal
**I**	A	0	0	1
	B	0	0	1
	C	0	0	0
**II**	A	1	1	1
	B	1	1	1
	C	1	0	1
**III**	A	0	0	1
	B	1	1	1
	C	0	0	0
**IV**	A	3	3	3
	B	3	3	3
	C	2	2	3

Groups I and III showed no histological signs of bone infection; group II showed a mild inflammatory reaction and group IV showed a severe osteomyelitis in all cases.

### Microbiological analysis

**Figure 8:**
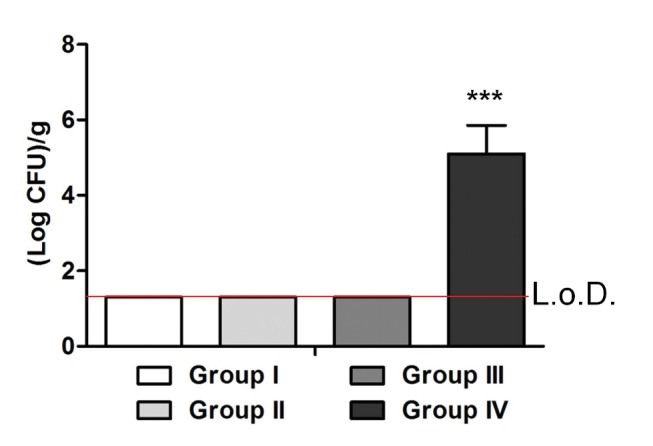
Bacterial load in bones of all experimental groups. The histogram compares the bacterial counts in the infected and control mice. After inoculation with an infecting dose of 1×10^3^ CFU/mouse, no colonies were detected in groups I, II and III. In contrast, a mean of 5.1±1.5 (Log CFU)/g of bone in the femoral canal was found in group IV (L.o.D.  =  limit of detection), which was statistically different from the other groups, as analyzed with one-way ANOVA and Bonferroni’s post-hoc (***P<0.0001; n = 4).

Microbiological analysis confirmed the histological findings. After sonication, no bacterial growth was observed either in control groups (I and III) or in group II. In contrast, great amounts of *S. aureus* were recovered from the samples collected from group IV (mean 5.1±1.5 (Log CFU)/g bone) (Fig. 8).

### Scanning electron microscopy

**Figure 9:**
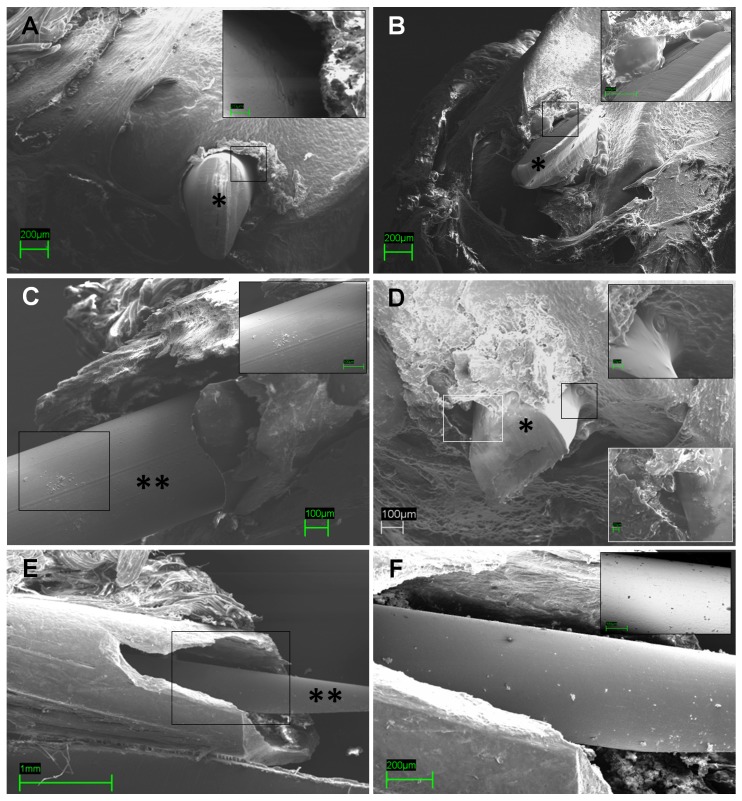
Representative images of absence of biofilm deposition within the implant site in groups I, II and III by Scanning electron microscopy (SEM) analysis. Absence of biofilm formation or bacterial deposition both on the cut ends of the implants (*) within the intercondylar notches in the control groups, precisely in group I (A, scale bar 200 µm, small box 20 µm) and group III (B, scale bar 200 µm, small box 100 µm), and along the implant length within the femoral canal (**) (C, scale bar 100 µm, small box 100 µm). Similarly, the absence of biofilm is shown in group II on the cut ends of the implants (*) (D, scale bar 100 µm, small boxes 20 µm) and along the implant length (**) (E, scale bar 1 mm; F, scale bar 200 µm, small box 100 µm).

**Figure 10:**
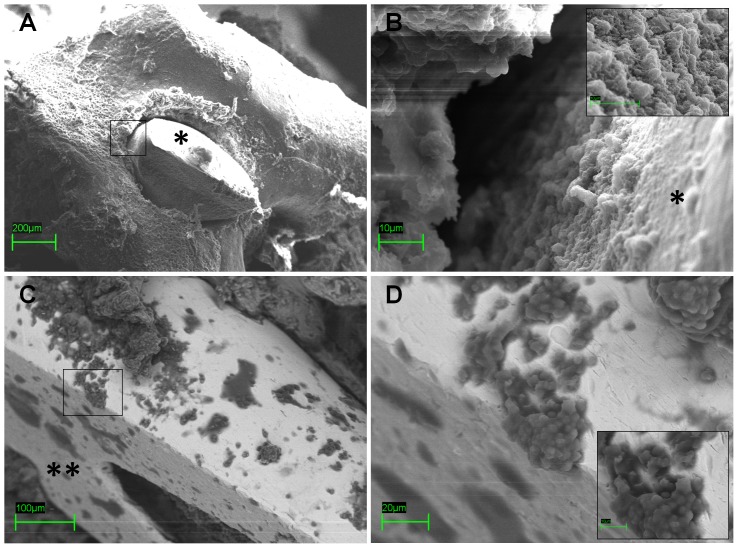
Representative images of presence of biofilm deposition within the implant site in group IV by Scanning electron microscopy (SEM) analysis. Presence of biofilm deposition on the cut ends of the implants (*) within the intercondylar notch (A, scale bar 200 µm), the implant-bone interface in the boxed area (B, scale bar 10 µm), and the opposite edge of the implanted nail (**) within the medullary canal (C, scale bar 100 µm) in the boxed area (D, scale bar 20 µm, small box 10 µm) are shown in group IV.

To determine whether biofilm formed on the implants in our mouse model, one implant per group was harvested at day 28 (Fig. 9 – 10). Microbial biofilm formation was examined under SEM after dehydrating and sputter-coating the samples with a conductive film.

No detectable biofilm formation or bacterial aggregates were visualized on the cut end of the implants within the knee joint both in control groups (group I and III) (Fig. 9 A, B) and in group II (Fig. 9 D). Similarly, no biofilm deposition was present along the implant length within the femoral canal neither in the control groups (Fig. 9 C) nor in group II (Fig. 9 E,F).

Only the samples from the infected NOD/ShiLtJ mice (group IV) showed prominent biofilm growth around and on the cut end of the implants (Fig. 10 A, B) and along the implant length fixed within the femoral canal (Fig. 10 C, D).

## Discussion

To our knowledge, this is the first description of an animal model of orthopaedic implant-related infection in diabetes.

Postsurgical infection after prosthetic surgery is a severe complication that often requires additional surgery and prolonged antibiotic treatments, with diminished quality of life and added costs. Bacterial adherence to foreign implanted materials and subsequent microbial biofilm formation are distinctive characteristics of implant-associated infections [1], [27]. Treatment of these infections poses medical and surgical challenges with a higher risk of morbidity and poor clinical outcome [5], [28], [29]. When left untreated, serious infections can progress to osteomyelitis and bone destruction [30].

Although the type of casual microorganism and its antibiotic resistance are critically important in the treatment and the prognosis of septic complications in orthopaedics [31], host type is probably even more important as a predictive factor of implant-related infection and the relative chance of clearing it [32], [33]. *S. aureus* ATCC 25923, a common clinical isolate strain, can produce biofilm in vitro [25]. This strain has also been described to reproduce the periprosthetic infections in many animal models [1], [34], [35].

In this regard, diabetes, because of its increasing prevalence worldwide, is emerging as prominent risk factor of staphylococcal infections and implant-related infection in orthopaedics [9], [10].

To investigate host response to infection and ensuing complications in diabetics as compared to healthy patients, and with a view to eventually employ our model to compare pharmacological and surgical strategies for infection control in orthopaedics, we developed an implant-related *S. aureus* infection model in the non-obese diabetic mouse (NOD/ShiLtJ) in which we compared the host response to identically infected healthy animals, CD1 mice in this model. The NOD/ShiLtJ mouse provides a good model because it spontaneously develops autoimmune diabetes and exhibits immunological and clinical similarities to type I diabetes in humans [10], [36]. The diabetic mouse model of implant-related bacterial infection has unique features that offer a valid option to study the effect of diabetes on nosocomial infections comparable to that any other laboratory animal could satisfy, except perhaps for the diabetic rat [37]. Our model can also be successfully employed to evaluate bacterial load and bone response in infected limbs, as previously described in non-diabetic mice [22], [24], [27], [38]. In addition, permits evaluation of infection-induced osteomyelitis through the direct measurement of the host response in infected animal bones by combining the use of imaging techniques with clinical, microbiological and histological analyses, which confirmed the validity of this animal model.

In the present study, the changes in body weight correlated with induction and response to infection. The weight loss in the infected CD1 mice during the early stage of infection (7 days post-inoculation) could be closely related to the time required for immune cell activation and for mounting activity against bacteria. In fact, 7 days post inoculation, the CD1 mice were able to fight against infection and regain their body weight, as previously reported for C57BL/6J mice [39]. Differently, however, the diabetic mice were unable to clear the infection over time and continued to lose weight or regained it very slowly. This phenomenon is linked to defects in the bactericidal activity of polymorphonucleated cells in type 1 diabetic individuals, as reported elsewhere [10], [40]–[42] and to the direct inhibitory effect on the adaptive immune system which decreases proliferative response and delays the hypersensitivity reaction of T-cell function in diabetic patients [43]–[44].

Moreover, the higher basal WBC values observed in the diabetic mice has also been reported in obese patients and in patients with impaired glucose tolerance [45], in whom the increased WBC count is thought to be responsible for macro- and microvascular complications [46]. The relative increase in total WBC count in the infected diabetic mice (group IV) was higher than in the other experimental groups 4 weeks post inoculation, suggesting that the infection due to *S. aureus* was maintained during the chronic phase, as also demonstrated by Pettersson et al. [47].

CRP, a well-known acute phase protein in humans and frequently used as a sensitive diagnostic marker for inflammatory response, was analyzed 28 days after surgery. All serum levels obtained in our study are underestimated, precluding comparison with serum CRP values in humans. In humans, serum CRP concentrations generally increase from <1 µg/ml up to 500 µg/ml during inflammation, whereas, in mice, serum CRP is <2 µg/ml, as found in our study (mean <2 µg/ml), and does not usually increase during the acute phase response [48]. Furthermore, the normal CRP concentration in mouse serum has not yet been established [48].

Typically, periosteal thickening and elevation are the earliest radiographic signs of osteomyelitis in humans and are followed by osteolytic changes related to subacute or chronic osteomyelitis [49]. In the present study, micro-CT showed distinct changes within the bone structure due to the infection in the *S. aureus*-inoculated diabetic mice, confirming the development of chronic osteomyelitis.

Histopathological determinations confirmed these results showing more pronounced signs of infection in the infected diabetic mice (group IV) as compared to the other groups. Histopathological signs of infection were present in all diabetic mice with a positive bacterial culture. As expected, in the uninfected groups (I and III) but, unexpectedly, also in group II, signs of infection were absent. Our findings are in line with those of other studies, which attributed cortical bone resorption by osteoclasts, medullary canal microabscesses and periosteal fibroblast proliferation during the inflammatory chronic phase to activation of polymorphonucleated cells [10], [38], [50].

These histological findings corroborate our imaging data, demonstrating inflammatory cell infiltration and bacteria in both the knee joint and the bone near the implant in group IV in particular. Although inflammatory cells were abundant at the prosthetic implant site, defects in the bactericidal activity of the immune system, as reported in diabetic patients, may explain the poor reaction toward *S. aureus* infection in the diabetic mice [51], [52].

The clinical, histological and imaging analyses of signs of infection, and positive culture in agar plating of the sonicated bones and tissues from group IV demonstrated the accuracy of our diabetic model.

Interestingly, all the samples harvested from the infected diabetic mice at 4 weeks post inoculation showed microbial biofilm formation, as detected on SEM analysis. SEM visualized the bacteria embedded in fibrotic tissue and biofilm growth on the implants after sputter-coating with a conductive film. These data, in spite of the limit related to the evaluation of a single sample per group, confirm that the model may be used to study biofilm formation in chronic post-arthroplasty infections [5], [29].

Finally, the microbiological analyses revealed marked bacterial growth in the diabetic mice, infected with an inoculum of *S. aureus* identical to that of the healthy CD1 mice [24], which recovered completely from the induced low-grade infection.

Our model is designed to approximate the clinical approach in which uncoated implants and ceftriaxone administered by a one-shot systemic injection are commonly used in the prophylaxis of orthopaedic infection [53], [54]. However, one limitation of the model is that no other antibiotics, posology or local injections were tested to compare reactions between the healthy and the diabetic mice, as instead other studies in non-diabetic laboratory animals have done [55]–[57]. And though overall successful, the results were obtained in a limited number of animals. Furthermore, in spite of the small size of mouse bones, we obtained excellent results with a 25G needle as a prosthetic implant, which will need to be verified with commonly used implant metals such as titanium or steel alloy. The differences regarding the genetic background between CD1 and NOD/ShiLtJ mice could affect their response to infection and could represent a limitation to this study, although, the use of CD1 mice as control for NOD/ShiLtJ is currently reported in many studies on diabetes [58]–[60]. Nevertheless, taken together, our data demonstrate the validity of our animal model for orthopaedic infections and osteomyelitis caused by bacteria commonly responsible for infections in diabetic patients.

## Conclusions

This original diabetic mouse model of orthopaedic implant-infection shows the different susceptibility of diabetic mice to local peri-prosthetic infection as compared to healthy individuals. The model offers a new, relatively inexpensive tool to investigate *in vivo* host response to different treatment options in diabetic and non-diabetic subjects. It also provides an opportunity to further study microbial biofilm formation in implant-related infections in diabetes.
